# Assessment of Blood Pressure Using Only a Smartphone and Machine Learning Techniques: A Systematic Review

**DOI:** 10.3389/fcvm.2022.894224

**Published:** 2022-06-13

**Authors:** Fridolin Haugg, Mohamed Elgendi, Carlo Menon

**Affiliations:** ^1^Biomedical and Mobile Health Technology Lab, ETH Zurich, Zurich, Switzerland; ^2^Department of Mechanical Engineering, Karlsruher Institute for Technology, Karlsruhe, Germany

**Keywords:** digital health, blood pressure, smartphone, hypertension, machine learning

## Abstract

Regular monitoring of blood pressure (BP) allows for early detection of hypertension and symptoms related to cardiovascular disease. Measuring BP with a cuff requires equipment that is not always readily available and it may be impractical for some patients. Smartphones are an integral part of the lives of most people; thus, detecting and monitoring hypertension with a smartphone is likely to increase the ability to monitor BP due to the convenience of use for many patients. Smartphones lend themselves to assessing cardiovascular health because their built-in sensors and cameras provide a means of detecting arterial pulsations. To this end, several image processing and machine learning (ML) techniques for predicting BP using a smartphone have been developed. Several ML models that utilize smartphones are discussed in this literature review. Of the 53 papers identified, seven publications were evaluated. The performance of the ML models was assessed based on their accuracy for classification, the mean error measure, and the standard deviation of error for regression. It was found that artificial neural networks and support vector machines were often used. Because a variety of influencing factors determines the performance of an ML model, no clear preference could be determined. The number of input features ranged from five to 233, with the most commonly used being demographic data and the features extracted from photoplethysmogram signals. Each study had a different number of participants, ranging from 17 to 5,992. Comparisons of the cuff-based measures were mostly used to validate the results. Some of these ML models are already used to detect hypertension and BP but, to satisfy possible regulatory demands, improved reliability is needed under a wider range of conditions, including controlled and uncontrolled environments. A discussion of the advantages of various ML techniques and the selected features is offered at the end of this systematic review.

## 1. Introduction

Hypertension, also known as high blood pressure (BP), is a major global public health issue and a “silent killer”, according to the World Health Organization (1). Elevated BP disproportionately affects populations in low- and middle-income countries where health systems are underdeveloped. It can cause heart disease, stroke, kidney failure, premature mortality, and disability. Unfortunately, in many cases, high BP is asymptomatic; thus, it is often undiagnosed because patients do not have access to proper equipment or it is too inconvenient for them to measure BP regularly.

If BP is measured at home, it is usually done with a cuff. A BP cuff is a medical device that is wrapped around a patient's arm and then inflated in order to measure BP. Typically, BP classification follows the ISH 2020 recommendations for a single measurement with a validated upper arm-cuff device (2). The cuff-based application is considered by many patients to be uncomfortable and clumsy. The photoplethysmography (PPG) technology is a promising solution, as it is a simple, easy to use, and convenient method (3). The use of PPG signals could increase the number of measurements taken throughout the day, which might increase the likelihood of early detection of hypertension (4).

Smartphones could address this need and become an easily accessible alternative to BP cuffs. Many people rely on smartphones every day and are familiar with their use. Smartphones have a variety of sensors, such as cameras, microphones, light emitters, and force sensors, that can be used to detect cardiovascular signals. These signals can be used to estimate BP. In addition to or independent of the sensors, a machine learning (ML) model could be implemented to estimate BP using typical risk factors. Common indicators associated with hypertension include age, gender, smoking, body mass index (BMI), obesity, stress, cholesterol level, physical activity, lipoprotein levels, and family history (5).

In the current literature, some reviews have evaluated the assessment of BP using ML, smartphone or both. For example, Steinmann et al. (6) provided an overview of how video cameras and smartphones could measure BP non-invasively. Hosanee et al. (7) evaluated the literature that assesses the reliability of single-site PPG-based approaches for BP monitoring. Martinez-Rios et al. (8) summarized ML models that use the combination of clinical and sociodemographic data (e.g., age, gender, and BMI) or physiological signals (e.g., ECG and PPG). In the current review, the focus is on ML models and a selection of smartphone features for early hypertension detection. The features included smartphone sensors and questionnaires completed by patients; as such, this review evaluates the utility and accuracy of these features in detecting BP.

## 2. Methodology

### 2.1. Search Strategy

This review was conducted in accordance with the recommendations of the Preferred Reporting Items for Systematic Review and Meta-Analysis (PRISMA) guidelines (9). Four different databases were used for the literature search: Institute of Electrical and Electronics Engineers (IEEE Xplore), PubMed, Excerpta Medica Database (Embase), and Google Scholar. IEEE Xplore is a research database that allows users to find and access journal articles, conference proceedings, and other publications in the subjects of computer science, electrical engineering, and electronics, as well as other related fields. It primarily contains content released by the IEEE and other collaborators. The PubMed database is maintained as part of the Entrez system of information retrieval managed by the United States National Library of Medicine at the National Institutes of Health; it contains publications about life sciences and biomedical topics. Embase, produced by Elsevier, is a biomedical and pharmacological bibliographic database of published literature. Google Scholar is a search engine that is used for general literature searches of scientific documents.

The four databases provided the following results for papers published from January 1, 2012 to January 1, 2022. On IEEE Xplore, eight articles were identified with the search terms: (“Document Title”: blood pressure OR “Document Title”: hypertension) AND (“Full Text & Metadata”: smartphone OR “Full Text & Metadata”: smart phone) AND (“Full Text & Metadata”: machine learning OR “Full Text & Metadata”: AI OR “Full Text & Metadata”: data driven). On PubMed, 14 articles were identified with the following search terms: (blood pressure[Title] OR hypertension[Title]) AND (smartphone OR smart-phone) AND (machine learning OR AI OR data driven). On Embase, 11 articles were retrieved using the following search terms: (“blood pressure”: ti OR “hypertension”: ti) AND (“smartphone” OR “smart phone”) AND (“machine learning” OR “AI” OR “data driven”). Google Scholar offers very limited advanced search options, therefore the keywords were changed and only the 30 most relevant results evaluated. For Google Scholar the following search terms were used: “blood pressure” OR “hypertension” AND smartphone AND machine learning.

### 2.2. Performance Metrics

To compare the studies, the performance metrics were selected in advance. First, classification accuracy (ACC) is a performance metric specified for all the classifier models in this review. It describes the ratio of the number of correct predictions to the total number of input samples. The percentage of true positive cases correctly classified is referred to as sensitivity and the percentage of real negative data samples correctly classified is referred to as specificity. The ratio of true positives to true positives plus false positives is known as precision. The recall and precision scores are used to generate the F1-score. Cohen's kappa is used in classification as a measure of agreement between observed and predicted classes; the simplified equations are shown in Table 1. The Receiver Operator Characteristic (ROC) curve is a binary classification evaluation metric. It is a probability curve that compares the true positive rate to the false positive rate at different thresholds. The Area Under the Curve (AUC) is a summary of the ROC curve that measures a classifier's ability to distinguish between classes. The AUC value ranges from 0 to 1, with 1 denoting a perfect classifier and 0 denoting a poor classifier.

**Table 1:** Performance metrics used to examine the performance of ML models for regression and classification problems.

**Type of task**	**Performance metric**	**Equation**
Classification	Accuracy (ACC)	$\frac{TP+TN}{TP+TN+FP+FN}$
Classification	Sensitivity	$\frac{TP}{TP+FN}$
Classification	Specificity	$\frac{TN}{TN+FN}$
Classification	Precision	$\frac{TP}{TP+FP}$
Classification	F1-Score	$\frac{TP}{TP+\frac{1}{2}\left(FP+FN\right)}$
Classification	Kappa for binary classification	$\frac{2\left(TP\cdot TN-FN\cdot FP\right)}{\left(TP+FP\right)\left(FP+TN\right)+\left(TP+FN\right)\left(FN+FN\right)}$
Regression	Mean absolute error (MAE)	$\frac{1}{n}\overset{n}{\underset{i=1}{\sum }}|x_{i}-y_{i}|$
Regression	Mean error (ME)	$\frac{1}{n}\overset{n}{\underset{i=1}{\sum }}x_{i}-y_{i}$
Regression	Standard deviation of error (SDE)	$\sqrt{\frac{1}{n-1}\overset{n}{\underset{i=1}{\sum }}{\left[\left(x_{i}-y_{i}\right)-\text{ME}\right]}^{2}}$

*FN, false negative; FP, false positive; n, number of samples; TN, true negative; TP, true positive; x_i_, estimated value; y_i_, true value*.

For regression, the mean error (ME) and standard deviation of error (SDE) were the designated performance metrics. The SDE is a measure of dispersion or how widely the values are spread out. The ME is the mean error in a set of estimates with consideration of their direction. Mean absolute error (MAE) calculates the average magnitude of errors in a set of estimates without considering their direction.

## 3. Results

### 3.1. Search Strategy

**Figure 1:**
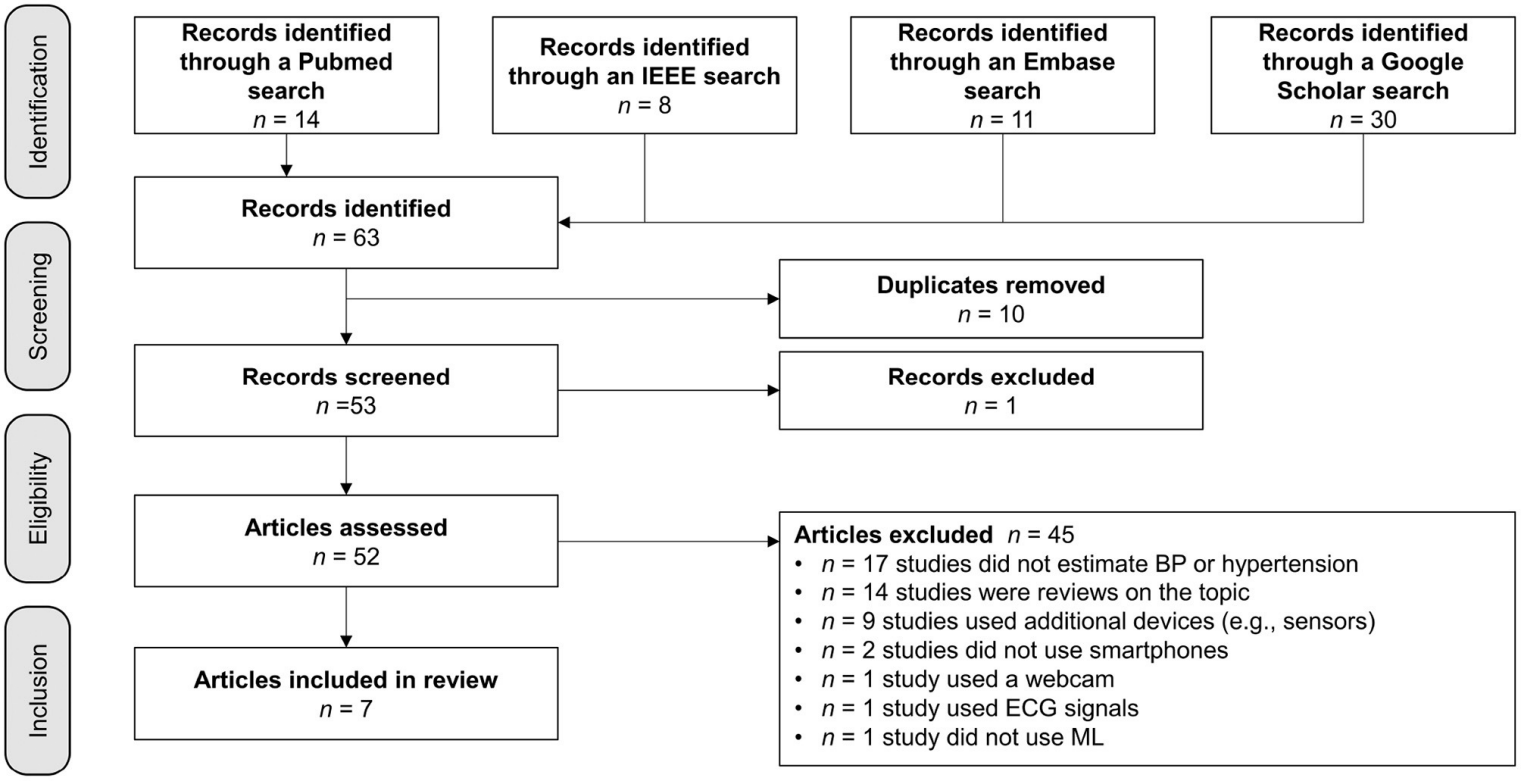
Search strategy: Filter for relevant publications.

During the screening process, 10 duplicates and one article that did not fit the intended format were eliminated. Next, 52 articles were evaluated for eligibility, and any publications that were not specifically about measuring BP with a smartphone were eliminated. Nine articles were removed because other devices or more than one smartphone were used to measure BP, as shown in Figure 1. The search resulted in the exclusion of 14 reviews, 17 papers were deemed non-applicable, since they did not focus on BP measurement. Two studies were excluded because they did not use smartphones. One was excluded because the authors used a webcam and another was removed because the study team used an electrocardiogram (ECG) signal. Instead of ML, one research group applied a mathematical model. The detailed review summarizes the essence of the seven articles selected based on criteria-based filtering.

### 3.2. ML in BP Assessment: Models, Datasets, and Features

**Figure 2:**
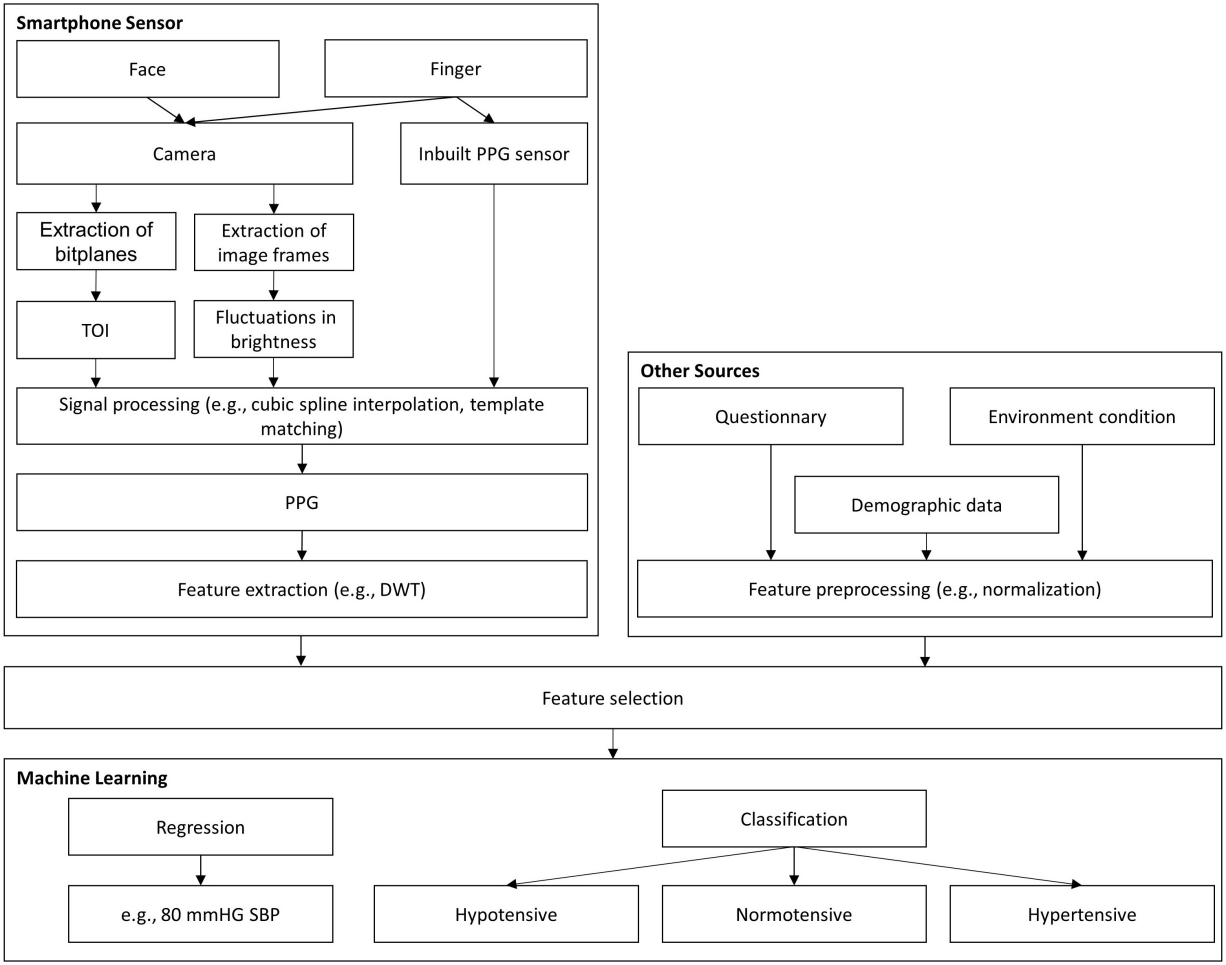
Graphical abstract for BP assessment. DWT, discrete wavelet transformation; PCA, principal component analysis; PPG, photoplethysmography; TOI, transdermal optical imaging; SBP, systolic blood pressure.

Two approaches were used to investigate the detection of hypertension and BP monitoring using a smartphone and ML models, as shown in Figure 2. The first approach used inputs derived from smartphone sensors, such as the camera, and, if possible, further input features, such as demographic data. The second approach consisted of estimating individual hypertension risk scores ascribed to demographic data or other sources (e.g., activity behavior), which were obtained by questionnaires on the smartphone.

These models can be divided into classification and regression tasks, as shown in Figure 2. With classification, the aim is often to detect the level of hypertension risk. Regression models are usually employed to estimate BP with continuous values. Although the two models have the same purpose, they have different evaluation criteria, making comparisons challenging. Dealing with outlier data and unbalanced datasets are among the additional challenges related to ML models. Therefore, the use of comment preprocessing steps in dealing with outliers is also highlighted in this review.

#### 3.2.1. ML Models Based on PPG Signals

A common method for calculating BP is to extract the features related to the pulse shape from the pulse waveform. Most smartphone technologies use cameras to extract PPG signals from the light reflected on the skin. A comparison between all ML models discussed in the included studies is demonstrated in Table 2.

**Table 2:** Summary of ML models based on PPG signal.

**References**	**Year**	**Participants**	**Gender**	**Normotensive and hypertensive**	**Features**	**ML Model**	**Recording duration**	**Gold standard**	**Performance metrics**
Luo et al. (10)	2019	1,320 adults	59% male 41% female	Only normotensive	126 features from face transdermal blood flow, 29 features relating to room temperature and demographic data (after PCA, only 30 eigenvectors)	ANN	2 min	Cuff-based	ME ± SDE −0.20 ± 6.00 mmHg for DBP 0.39 ± 7.30 mmHg for SBP
Dey et al. (11)	2018	205 adults	44% male 56% female	Normotensive and hypertensive	233 features extracted from the PPG signal, (trained different LR models each for different demographic groups)	LR	15 min	Cuff-based	MAE ± SDE 5.0 ± 6.1 mmHg for DBP 6.9 ± 9.0 mmHg for SBP
Gao et al. (12)	2018	65 adults	62% male 38% female	Only normotensive	Systolic upstroke time and diastolic time, demographic data (age and gender), 13 to 22 features from DWT	SVM	2 min	Cuff-based	ME ± SDE 4.6 ± 4.3 mmHg for DBP 5.1 ± 4.3 mmHg for SBP
Gaurav et al. (13)	2016	3,000 subjects	N/R	Only normotensive	46 all based on PPG	ANN	N/R	Invasive	ME ± SDE 0.03 ± 4.72 mmHg for DBP 0.16 ± 6.85 mmHg for SBP
Visvanathan et al. (14)	2016	17 subjects	N/R	Normotensive and hypertensive	14 features directly from PPG, demographic data (age, weight, and height)	SVM	N/R	Cuff-based	ACC 99.29% for DBP ACC 100% for SBP

*ANN, artificial neural network; DBP, diastolic blood pressure; DWT, discrete wavelet transformation; MAE, mean absolute error; ME, mean error; N/R, not reported; PPG, photoplethysmography; SVM, support vector machine; LR, Lasso regression; SBP, systolic blood pressure; SDE, standard deviation of error*.

Gaurav et al. (13) proposed an artificial neural network (ANN) regression model to determine BP. The dataset contained 3,000 records captured with a PPG sensor from the Samsung Galaxy Note 5. The records were divided into windows between two valleys in the PPG signal. Inconsistent windows were removed from the records, followed by min–max normalization. From one window, eight features were extracted from the PPG signal; 19 features were extracted from the second derivative of the PPG signal, along with eight non-linear cardiac cycle time ratio-based features. Eleven features from heart rate variability were extracted by considering 10 consecutive peak intervals of the PPG signal. In total, Gaurav et al. (13) considered 46 features to determine diastolic blood pressure (DBP), all derived from the PPG signal for the ANN.

To determine systolic blood pressure (SBP), Gaurav et al. (13) also used the DPB value as an input feature. The final output was determined by combining the weighted results of three models, each with four hidden layers. The ME ± SDE achieved by their suggested method was 0.03 ± 4.72 mmHg for DBP and 0.16 ± 6.85 mmHg for SBP. The MAE achieved was and 3.21 mmHg for DBP and 4.47 mmHg for SBP. To evaluate the performance, 20% of the data was used for testing. However, high accuracy was achieved only with a dataset that did not include PPG signals from subjects with hypertension.

The Gaurav et al. (13) model achieved remarkable accuracy with input features that were only based on the PPG signal. Other studies have combined the features of the PPG signal with other input features, such as demographic data. For example, Visvanathan et al. (14) added demographic features to a PPG-based approach to improve accuracy. The PPG signal was captured with the fingertip placed on a smartphone camera with the LED flashlight turned on. The peaks and valley points of every cycle were detected using a simple peak detection code. For each cycle, 14 features were calculated. Furthermore, each candidate's height, weight, and age were used as input features. Altogether, 17 features served as inputs for the support vector machine (SVM) to predict BP levels. The model had five output classes, ranging from very low BP to very high BP. The SVM achieved an accuracy of 99.29% for DBP and 100% for SBP, with a dataset of 512 preprocessed PPG samples from 17 subjects. The hold-out method was used to evaluate the performance. In the preprocessing step, a finite-state machine model was used to reject noise. This study showed that the performance of the model could be increased by adding demographic data.

Gao et al. (12) also collected 78 PPG signals from 65 subjects by placing their fingertips on a smartphone camera. A 2-min signal was recorded, but only 1 min was used for features extraction. Every subject had a normal BP. As an input feature, systolic upstroke time and diastolic time, along with gender, age, and discrete wavelet transform (DWT) coefficients, were used. The systolic upstroke time and the diastolic time coefficients were obtained on the continuous wavelet transform with a Mexican hat wavelet smoothed series using the MinDetect and MaxDetect functions in Mathematica. The best DWT coefficients were determined using automated feature selection, which stops adding features when none of the alternatives improves upon the merit of a current feature subset. The SVM that was used with a radial basis function kernel achieved an ME ± SDE of 5.1 ± 4.3 mmHg for SBP and 4.6 ± 4.3 mmHg for DBP. The performance was evaluated with 10-fold cross validation.

While the authors of the two studies discussed above used analogous approaches, unfortunately the different performance metrics of ACC and ME ± SDE made it extremely difficult to compare the models and determine which input feature selection was best.

Other studies have facilitated this comparison. Dey et al. (11) recorded PPG signals using the heart rate sensor included in Samsung Galaxy S6 phones. The signal was recorded for 15 min for each subject. Data were collected from 205 subjects with diverse profiles. A variety of prepossessing steps were applied involving wavelet smoothing, followed by a trend removal and dynamic peak search on the original signal and the inverted signal to obtain minima and maxima. Furthermore, template matching was performed with cubic spline interpolation and min–max normalization. A total of 233 features were extracted from the PPG signals in the time and frequency domains, while three demographic features (age, gender, and BMI) served as input features for the ML model. Age, gender, and BMI were not elements of the input vector for the ML model; instead, the dataset was divided into categories, such as male and female. For each subgroup, an ML model was trained. Several ML models were tried; the best performing model was the Lasso regression (LR) model, with an MAE of 6.9 mmHg for SBP and 5 mmHg for DBP. The hold-out method was applied to evaluate the model's performance, with 17% of the data used for testing. This ML model also achieved an SDE of 9.0 mmHg for SBP and 6.1 mmHg for DBP. Dey et al. (11) also provided a working smartphone application called InstaBP.

It is possible to compare the approaches used by Gao et al. (12) and Dey et al. (11) since they are rather similar. Consequently, the features chosen by Gao et al. (12) and their non-linear regression technique seem to be better in terms of SDE.

Alternatives to the fingertip approaches presented in these papers were investigated in other studies. For instance, hemoglobin signals can be obtained by a smartphone using facial recognition technology. An elaborate study in this vein was conducted by Luo et al. (10) who examined the faces of more than 1,328 subjects. Transdermal optical imaging (TOI) was used to estimate BP. The blood volume pulse was retrieved from 17 face regions using an ML model that selected the bit planes from a 2-min video with the greatest signal for hemoglobin.

Each subject was seated when the video was recorded on an Apple iPhone 6, and the environment was strictly controlled to avoid noise. A total of 155 features were used. The first 126 of these features were derived from the face transdermal blood flow data collected from the subject's 17 areas of interest, such as pulse amplitude, pulse amplitude in the heart rate band, pulse rate, pulse rate variability, pulse transit time, pulse shape, and pulse energy. The remaining 29 features consisted of meta-features that helped normalize the varied imaging settings, as well as features relating to the subject's room temperature and physical attributes (e.g., age, weight, and skin color). Principal component analysis decreased the data dimensionality, resulting in 30 eigenvectors that were input into an ANN to determine BP. The model achieved an ME ± SDE of 0.39 ± 7.30 mmHg for SBP and −0.20 ± 6.00 mmHg for DBP. The hold-out method with 15% of the data used for testing was applied to evaluate the model's performance. The high accuracy is impressive in this case, and it seems to be pointing to this feature as a viable alternative to fingertip-based approaches. However, the selfie video was taken under ideal conditions. If the selfie video is recorded independently by smartphone users, significantly more noise is expected.

#### 3.2.2. ML Models Based on Demographic Data and Other Sources

Because it is possible to determine a hypertension risk score based on demographic data or other sources (e.g., age or weight), this section describes the methods in which the risk score was determined based on answers to a questionnaire on a smartphone or web-based applications.

For example, Fitriyani et al. (15) developed an ML model that used five features (age, weight, height, waist circumference, and hip circumference) to determine hypertension. The output was a binary classification of hypertension: yes/no. Because only clinical data were used, the preprocessing steps differed from those used in the previously described papers. To detect and eliminate outliers, an isolation forest algorithm was used. The next step was the implementation of the SMOTETomek method to balance the datasets in terms of the classifications of hypertension and non-hypertension. The ML model consisted of a combination of multiple classification algorithms. The best results were achieved by combining an ANN, SVM, and decision tree (DT) as first-level classifiers and logistic regression as a second-level classifier. This model obtained an ACC of 85.73% (precision: 93.57%, sensitivity: 84.89%, F1-score: 88.8%, AUC: 0.88) for the dataset used in Golino et al. (16) with 139 male subjects after removal of the outliers. ACC is the metric commonly used to evaluate classification models. The number of correct predictions divided by the number of input samples indicates the classification accuracy. The model's performance was assessed with 10-fold cross-validation. One strength of this study is that the complete model was successfully implemented in a mobile app, showing how early smartphone-based hypertension detection could work.

Other researchers have estimated hypertension risk using more features. For instance, in addition to demographic features, Seto et al. (17) used data from 2 days of 24-h dietary recall interviews (assessing the intake of various macro- and micronutrients); a questionnaire about diet, behavior, and physical activity; and a nine-item mental health depression screener. Based on these input data, different models were investigated. Random forest (RF) was the best-performing ML model for binary hypertension classification, with an accuracy level of 73% (sensitivity: 53%, specificity: 83%, kappa: 0.37). The hold-out method was applied to evaluate the model's performance, with 25% of the data used for testing. The model was fitted using data from the 2015 to 2016 National Health and Nutrition Examination Survey (NHANES) conducted in the United States (18). The dataset included information on 5,992 adults. After filtering, incomplete entries were deleted from the set. One result of the study was a working web interface, which allowed for the development of a hypertension risk estimation scale by assigning a score to the answers for some of the questions.

In comparison to Seto et al. (17) and Fitriyani et al. (15) achieved greater accuracy using fewer input features. However, it is important to note that Seto et al. (17) used a significantly larger dataset and rigorous testing. Since both studies used data-driven models, the size of the corresponding dataset is very important. The comparison is presented in Table 3.

**Table 3:** Summary of the ML models using demographic data and other sources.

**References**	**Year**	**Participants**	**Normotensive and hypertensive**	**Features**	**ML model**	**Gold standard**	**Error**
Seto et al. (17)	2020	5,992 adults	67% normotensive and 33% hypertensive	This study included 125 features, including
demographic data, intake of macro- and
micronutrients, nutrition and physical activity behavior, and a depression screener	RF	N/R	ACC 73% Sensitivity 53% Specificity 83% Kappa 0.37
Fitriyani et al. (15)	2019	139 male adults	73% normotensive and 27% hypertensive	This study included five features, all of which are demographic data: age, weight, height, waist, and hip	Ensemble Learning (ANN, SVM, DT)	Cuff-based	ACC 86% Precision 93.57% Sensitivity 84.89% F1-score 88.8% AUC 0.88

*ACC, accuracy; ANN, artificial neural network; DT, decision tree; N/R, not reported; PPG, photoplethysmography; RF, random forest; SVM, support vector machine*.

#### 3.2.3. Datasets

In this review, three ML models for classification were presented, two of which used public datasets. In addition to the number of subjects in the dataset, the distribution of the classes of hypertension and normotension is also significant. Because most ML models for classification are built to enhance accuracy, imbalanced classifications represent a difficulty in predictive modeling. Consequently, the models may have poor prediction accuracy, particularly for minority classes. This is a dilemma because the minority class, in our case people with hypertension, is typically more significant than the majority class, so the problem is more susceptible to categorization errors for the normotensive class than for the hypertensive class. Furthermore, ML models are data-driven and rely on large datasets. Based on its pure sample size and the large number of possible input features, the 2015–16 NHANES (18) is a well-seeded dataset for ML models.

For regression ML models, there are related problems with the poor distribution of input data. This is why some researchers have limited themselves to the prediction of normotensive subjects. It is important to note that the non-public dataset of Luo et al. (10) with 1,328 normotensive subjects, was created with a considerable amount of effort, as a selfie video had to be taken and labeled with the given blood pressure for each person under controlled environmental conditions.

#### 3.2.4. Ground Truth Blood Pressure

Ground truth BP has mostly been determined with cuff-based approaches. Information provided by direct measurement that is known to be real or true is known as ground truth. Two of the three classification models used cuff-based BP measurements as the gold standard; only the NHANES (18) dataset use by Seto et al. (17) includes clinical diagnosed hypertension. The cuff-based technique is regularly utilized in outpatient clinical settings where hypertension diagnoses can be made; therefore, this method of measurement makes sense. Consequently, using cuff-based measurements as a reference could provide information on how smartphone-based approaches compare to the method currently utilized to diagnose hypertension in clinical outpatient settings.

## 4. Discussion

This systematic literature review addresses the current use of smartphones and ML models to assess BP and hypertension. Wearables, such as smartwatches or similar devices, were not covered in this study, despite their growing importance in the field of BP monitoring. Smartwatches can collect myriad points of data and are particularly promising for ML applications. PPG-based approaches are applied in the majority of smartphone methods for predicting BP.

Over the past five years, ML approaches for BP estimates and hypertension stage classifications with a smartphone have piqued the interests of researchers, as shown in this review. For ML regression models, it is beneficial to combine PPG signals and demographic data. Here, the literature is very clear and consistent. For demographic input data, age and BMI are generally used. Based on the studies analyzed in this review, a variety of ML models could be observed, yet no obvious preference for one model over another could be detected. Many small, non-public datasets make it particularly difficult to compare different approaches. It would be useful to develop a common public dataset as a benchmark that can be applied when comparing different ML models.

In this review, a variety of possible input features for an ML model were introduced. Anything related to BP that a user can answer with a simple questionnaire should also be investigated as a possible input feature for PPG-based models. It is possible that additional features, such as hip circumference measurements or data from a symptom questionnaire, might improve the performance of an ML model. Nevertheless, there is much disagreement on how to use PPG as input for the ML model, with a slight trend toward extracting few features from the PPG signals However, it is not possible to determine the methods by which the features are optimally extracted from the PPG signal because they are only one of several factors that influence performance of an ML model.

We also surveyed the primary metrics used to evaluate model performance. When detecting hypertension or classifying it into different stages, the most common metric was accuracy. When estimating BP values *via* regression models, the evaluation was mainly based on the MAE as well as the ME ± SDE. When the interpretability of the results is important, MAE is the measure of choice because it uses the same units as the estimated value (mmHg).

Despite these findings, it is difficult to evaluate whether classification or regression models are preferable for BP evaluation. Classification models have the advantage of being better at dealing with uncertainty because they do not have to estimate a continuous value. In this study, classification models without PPG signals were shown to be successful for estimating hypertension. PPG signals and demographic data were used as input characteristics in the classification algorithm proposed by Visvanathan et al. (14), which resulted in an extraordinarily high ACC score for hypertension prediction. In contrast, regression models frequently included only normotensive subjects, whereas all the classification models included hypertension patients. In many circumstances, simply predicting the cohort's BP value using the average BP of a similar group would result in a low error rate. If the goal is to diagnose hypertension on a smartphone as soon as is feasible, classification models are preferable. Furthermore, the two classes, hypertension and normotension, are likely to be easier for most people to comprehend than a continuous BP value.

It is worth noting that the 2018 Universal Standard for the Validation of Automated Blood Pressure Measurement Devices used in several studies, which was developed by the American Association for the Advancement of Medical Instrumentation (AAMI), the European Society of Hypertension, and the International Organization for Standardization, is inappropriate for such devices, particularly those requiring calibration (19). These protocols are intended for automated cuff BP devices that only monitor blood pressure for a single instant.

Several published methods have a ME ± SDE within the clinically acceptable range of 5 ± 8 mmHg (19). All four of the ML regression models in this review meet the AAMI standard for DBP. Two of the four ML regression models meet the same standard for SBP. Moreover, the study must be conducted with at least 85 subjects. Three of the four regression ML models in this review meet the minimum number of subjects criterion.

The mean absolute difference (MAD) from the IEEE Standard for Wearable, Cuffless Blood Pressure Measuring Devices (20) is similar to the MAE, with the difference that the reference BP is the average from two measurements. As defined in the standard, the MAD was not used by any of the papers in this review.

Five studies evaluated the model's performance using the hold-out method with 75–85% of the data used for training and 15–25% of the data used for testing. Two studies used cross-validation. The increased computational burden of running cross-validation is not a major concern for small datasets. A simple train-test split returns less trustworthy quality scores, so cross-validation is preferable for BP estimation with ML models.

Two out of those five studies reported recording a PPG/hemoglobin signal of 2 min; one study reported a PPG signal of 15 min. Two studies did not report the recoding length. No correlation could be found between the record length and the achieved performance. It is important to note that, for the feature extraction, usually only a window of the recorded signal is used.

Finally, there is a lack of studies on whether smartphones can record BP changes in real time, as would be triggered by, for example, participating in sports. A major issue with novel technologies is whether they can track BP changes, which are not assessed in the protocols for cuff devices. Furthermore, inter- and intra-individual BP variations are necessary for cuffless device evaluation, but they are challenging to acquire. Furthermore, the values from intra-arterial BP monitoring, which can track individual BP changes, are different from cuff-based measurements, which have been extensively used in clinical studies. Thus, the intra-arterial approach is impractical for clinical practice and academic research studies. Cuff-based BP measurement remains the gold standard method for BP measurement in clinical hypertension, and all guidelines are based on those measurements. Moreover, all the reviewed studies were calibration free.

Recommendations:

1. We recommend that ML models that use the PPG signal in combination with other input features, such as demographic data, always conduct an evaluation using only input features from the PPG signal to determine how well blood flow is detected and processed. The correlation between demographic data and BP can be very high, and the ML model that is used can achieve good results in small homogeneous groups without using the PPG signal. For example, in men, BMI was found to have a significantly weak negative correlation with both SBP and DBP levels (21). When demographic data and PPG features are used as input features, it is not possible to say how the features are weighted.
2. For the regression models, we propose using ME ± SDE as a performance metric. SDE provides an easy-to-comprehend picture of the error distribution. Furthermore, SDE has been employed in numerous investigations, making it easier to compare the findings reported in future studies. Because MAE utilizes the same units as the estimated value (mmHg), it is simple to understand and it should also always be reported.
3. We recommend using several performance metrics for the classification model. The ACC is useful for simple comparisons and it is easy to understand. However, especially for datasets with a large proportion of normotensive subjects, as was the case in this review, the ACC is not ideal. When accuracy is the objective, false positives and false negatives have the same costs. There is a simple approach to reducing the cost of an imbalanced dataset, such as one with 99% of the instances in one class and only 1% in the other. If the model only predicts the majority class, an ACC of 99% is achieved without any effort. The AUC is another metric that serves as a comprehensive indicator of ongoing changes in false positive and true positive rates. The AUC is a better performance metric and can partially solve the problems of the ACC, and it should always be reported as the performance metric for classification ML models (22).
4. To increase the performance of the ML model, we recommend considering all possible input features. Features from the PPG signal, as well as demographic data. Moreover, simple questionnaires about daily activity could be considered. It makes sense to take a top-down approach by first extracting all the possible features and systematically reducing them with, for example, a principal component analysis. Possible features to consider, in addition to those extracted from the PPG, include age, gender, weight, height, BMI, hip circumference, waist circumference, a daily activity questionnaire, or ambient conditions. It is not possible to determine in advance which combination of features will achieve the best performance.
5. To overcome the problem of too small a dataset with too few diverse subjects, we recommend using common resources to create a benchmark dataset that can be used to test different models. For example, a public dataset of selfie videos labeled with SBP and DBP would be suitable for this purpose.
6. We recommend focusing on the early detection of hypertension rather than estimating SBP and DBP values. In the near future, smartphones will not be able to replace clinical BP measurements their measurements are more susceptible to noise. However, if smartphones are used to detect possible hypertension in time to consult a doctor, this could help a large number of people and would be a major step forward in the fight against hypertension.
7. We recommend using cross-validation if the available computing power allows it. Cross-validation provides more trustworthy scores than a simple train-test split, especially for small datasets.
8. We recommend exploring optimal filters for iPPG signals and applying optimal filters used for non-smartphone-based PPG signals, such as the 4th order Chebyshev II filter (23).
9. There is a striking need for age diversity in evaluating iPPG-based technologies (24).
10. To ensure reliability and scalability, the technology needs to be tested on subjects with a diversity of skin pigmentation regardless of the racial or ethnic group (25).

## 5. Conclusion

It is possible to estimate BP using ML and a smartphone, and further investigation to improve the accuracy of such measures would be useful. The ultimate focus should be on the early detection of hypertension *via* a method that is accessible to most patients. It is conceivable that people could regularly check their hypertension risk score on their smartphone and, if there is a risk, follow up by getting a more precise measurement from a doctor. However, it does not seem plausible that clinical measurements will be replaced by those performed on a smartphone in the near future.

## Data Availability Statement

The original contributions presented in the study are included in the article/supplementary material, further inquiries can be directed to the corresponding author/s.

## Author Contributions

ME designed and led the study. FH, CM, and ME conceived the study. All authors approved final manuscript.

## Funding

Open access funding provided by ETH Zürich.

## Conflict of Interest

The authors declare that the research was conducted in the absence of any commercial or financial relationships that could be construed as a potential conflict of interest.

## Publisher's Note

All claims expressed in this article are solely those of the authors and do not necessarily represent those of their affiliated organizations, or those of the publisher, the editors and the reviewers. Any product that may be evaluated in this article, or claim that may be made by its manufacturer, is not guaranteed or endorsed by the publisher.
